# Investigation of MicroRNA in Mitochondrial Apoptotic Pathway in Systemic Lupus Erythematosus

**DOI:** 10.1155/2018/9026357

**Published:** 2018-07-10

**Authors:** Yu-Jih Su, Nai-Wen Tsai, Chia-Te Kung, Hung-Chen Wang, Wei-Che Lin, Chih-Cheng Huang, Ya-Ting Chang, Chih-Min Su, Yi-Fang Chiang, Ben-Chung Cheng, Yu-Jun Lin, Cheng-Hsien Lu

**Affiliations:** ^1^Department of Rheumatology, Allergy and Immunology, Kaohsiung Chang Gung Memorial Hospital and Chang Gung University, College of Medicine, Kaohsiung, Taiwan; ^2^Department of Neurology, Kaohsiung Chang Gung Memorial Hospital and Chang Gung University, College of Medicine, Kaohsiung, Taiwan; ^3^Department of Emergency Medicine, Kaohsiung Chang Gung Memorial Hospital and Chang Gung University, College of Medicine, Kaohsiung, Taiwan; ^4^Department of Neurosurgery, Kaohsiung Chang Gung Memorial Hospital and Chang Gung University, College of Medicine, Kaohsiung, Taiwan; ^5^Department of Radiology, Kaohsiung Chang Gung Memorial Hospital and Chang Gung University, College of Medicine, Kaohsiung, Taiwan; ^6^Department of Nephrology, Kaohsiung Chang Gung Memorial Hospital and Chang Gung University, College of Medicine, Kaohsiung, Taiwan; ^7^Department of Biological Science, National Sun Yat-Sen University, Kaohsiung, Taiwan; ^8^Department of Neurology, Xiamen Chang Gung Memorial Hospital, Xiamen, China

## Abstract

**Background:**

Accumulating evidence indicates that microRNAs play a pivotal role in the pathogenesis of systemic lupus erythematosus (SLE). This study tested the hypothesis that microRNA is associated with the mitochondrial apoptotic pathway in patients with SLE.

**Methods:**

Thirteen patients were in the clinical comparison study and microRNA study and overall 19 patients in the study of intracellular protein. Levels of microRNAs were determined by miRNeasy kit in 13 patients with SLE and 29 volunteer normal controls. Intracellular levels of caspase-9, caspase-10, MAVS, MDA5, and pIRF7 in mononuclear cells from 19 patiens and the SLE disease activity index (SLEDAI) were determined in all SLE patients. Correlation analyses were performed among microRNAs, intracellular adaptor proteins, and caspase levels and mean SLEDAI.

**Results:**

The ΔCT, defined by test reading difference between the target and the internal control microRNA (miR-451a), of miR-21-5p, miR-150-5p, and miR221-3p were significantly higher in plasma from SLE patients than in normal controls. miR-150-5pΔCT was positively correlated with both CRP and SLEDAI value. miR-150-5pΔCT was negatively associated with MAVS 70 kD. Caspase-10 protein levels were negatively associated with plasma miR-22-3pΔCT and miR-21-5pΔCT levels.

**Conclusions:**

Our study confirmed the hypothesis that these microRNAs were associated with the mitochondrial apoptotic pathway in SLE. miR-150-5pΔCT was positively associated with SLE disease activity and it was negatively correlated with MAVS 70 kD, which may facilitate viral survival and further enhance inflammation. On the other hand, miR-22-3pΔCT and miR-21-5pΔCT, were negatively correlated with caspase-10 levels, which may repress extrinsic apoptosis and increase cell survival.

## 1. Introduction

Systemic lupus erythematosus (SLE) is a chronic systemic disease affecting mostly women of child-bearing age. It is the prototype of autoimmune diseases because of the variety of its proposed pathogenesis mechanisms. Chronic or acute viral infection or reactivation is one of several important mechanisms involved in the pathogenesis of this condition [1–6]. Few markers reflect antiviral immunity clinically, with the exception of the antiviral immunoglobulins (e.g., IgG, IgA, or IgM). The peripheral blood mononuclear cells, PBMCs, include both lymphocytes and monocytes by definition. In SLE patients, these two leukocyte lineages are key players in disease pathogenesis and are key cells that fight viral infection. The major functions of these two leukocyte lines are antigen presentation and the execution of adaptive immunity and interferon production against infection [7, 8].

Aside from mononuclear cells of leukocytes, viruses play a role in inducing lupus and lupus flare-ups [4, 9–11]. In addition to the incorporation of the interferon pathway, we focused on antiviral molecules such as mitochondrial antiviral signaling protein (MAVS), melanoma differentiation-associated protein 5 (MDA5), and interferon regulatory factor 7 (IRF7) in this study. The postviral immune response should activate IRF genes [12]. Changes in IRF7 phosphorylation levels could be explained by aberrant activation of the NLRP3 pathway [13], STAT1 pathways [14], IRF3 [15], or downstream MAVS signaling due to inflammation. On the other hand, it might be caused by autoimmunity or cytokine milieu in SLE [16–18].

Levels of plasma microRNAs are deliberately controlled, requiring multiple layers of regulation involving the participation of various protein regulators and posttranscriptional modifications [19–23]. This study explored the associations between circulating microRNA and intracellular proteins involved in the mitochondrial apoptotic pathway including caspase, pIRF7, MAVS, and MDA5. Because of the possible benefits of choosing the appropriate immunosuppressant regimen, there is a need to improve our understanding of the clinical significance of antiviral immunity in SLE.

## 2. Patients and Methods

### 2.1. Study Patients

The patients with definitive diagnosis of SLE who were followed up at the Rheumatology Outpatient Clinic for more than six months were prospectively evaluated and compared to 29 healthy subjects. The diagnostic of SLE was based on the 1997 revision of the 1982 American College of Rheumatology classification criteria for SLE [24], and the assessment of SLE disease activity was based on the SLE disease activity index (SLEDAI) [25].

There were 19 SLE patients enrolled, and all patients did not undergo changes in steroid dose or immune-modifying medication during the study period. For comparison, 29 age- and sex-matched healthy subjects were enrolled as healthy controls. The individual plasma microRNA was retrieved in 13 SLE subjects, but the experiment from the rest of six SLE patients was suboptimal. In total, there were 13 patients accomplished in the plasma microRNA and clinical comparison study and 19 patients in the study of intracellular protein study.

The Institutional Review Committee on Human Research reviewed and approved the study protocol and all participants provided informed consent. Patients were excluded if they had autoimmune diseases other than SLE.

### 2.2. Clinical Assessments

All 19 subjects had complete medical examinations upon enrollment. Clinical data including complement levels and anti-double strand DNA levels were performed regularly and collected upon enrollment. Biomarkers, including demography data, complement levels, anti-ribosomal p autoantibody (a-rib p), anti-double strand DNA autoantibody (a-dsDNA) levels, and disease activity index were also collected.

### 2.3. Assessment of Protein Expression and MicroRNA Levels

#### 2.3.1. Western Blot Analysis

Levels of intracellular proteins, including MAVS (57 kD and 70 kD), pIRF7 (65 kD), caspase-9 (37 kD), caspase-10 (59 kD), and MDA5 (135 kD), were determined by western blotting. The MAVS were defined as the larger one (70 kD) and the smaller one, mini-MAVS (57 kD) [26–29]. Blood samples were collected by venipuncture of forearm veins of the 19 SLE patients. Peripheral blood mononuclear cell (PBMC) intracellular protein levels of phosphorylated interferon regulator factor 7 (pIRF7), mitochondrial antiviral signaling protein (MAVS), and melanoma differentiation-associated protein 5 (MDA5) were detected by western blotting. Detailed procedures were described in the previous study [30]. The reagents and antibodies were rabbit polyclonal antibodies recognizing caspase-9 (Cell Signaling, #9501), phospho-IRF-7 (Cell signaling, #5184), rat polyclonal antibodies recognizing caspase-10 (Biolegend, #645202), and anti-mitochondrial antiviral signaling antibody (MAVS) (Abcam #ab25084).

Caspase-9 activation was demonstrated by observing cleaved caspase-9 (active caspase-9, caspase-9c, 37 kD) from original caspase-9 (caspase-9, 47 kD) [18]. Caspase-10 activation was demonstrated by observing cleaved caspase-10 (active caspase-10, caspase-10c, 43 kD) from original caspase-10 (caspase-10, 59 kD) [19]. The MAVS were shown to have two types with similar activities: 70 kD (full-length MAVS) and 57 kD (mini-MAVS) [27, 29, 31, 32].

#### 2.3.2. MicroRNA Measurement

Blood samples were collected by venipuncture of forearm veins of the 19 SLE patients and 29 normal subjects. Samples were centrifuged at 1000×*g* for 10 min to pellet cellular debris. The supernatant was used for RNA extraction. Total RNA was extracted from 300 *μ*L of fluid using the miRNeasy kit (Qiagen) as described by Weber et al. [33]. Amplification was performed according to the manufacturer's instructions (Qiagen). We assessed the extracted RNA for quality and quantity using an Agilent 2100 Bioanalyzer and NanoDrop 1000 spectrophotometer (Thermo Scientific). For the bioanalyzer, the RNA 6000 Pico chip was used for quantification and an initial quality measurement, followed by the use of a Small RNA chip to gain a more detailed view of RNAs in the 6- to 150-nucleotide size range. We performed quantitative real-time PCR (qPCR) according to manufacturer's instructions (Qiagen) to profile the miRNA distribution in body fluid samples. In brief, 5 *μ*L total RNA was collected and pooled from the samples of the same fluid type, and the cDNA was produced using the miScript Reverse Transcription kit (Qiagen). We used the Matrix Hydra eDrop (Thermo Scientific) to mix the cDNA sample and the qPCR master reagent [Human miScript Assay 384 set v10.1 (Qiagen)] to reduce pipetting error. Any wells with multiple melting temperature values were excluded from further analysis. We also used individual Human miScript Assays to validate the 384 miRNA qPCR set. Data was analyzed using SDS Enterprise Database 2.3 (Applied Biosystems) and normalized to a global mean instead of specific miRNA or noncoding RNA signals.

### 2.4. Statistical Analysis

Data were expressed in the form of mean ± SD or median (interquartile range). Categorical variables were compared by Chi-square test or Fisher's exact test. Continuous variables were arcsine-transformed to improve normality, and then comparisons between two groups were performed using Student's *t*-test. Correlation analysis was used to explore the relationship between the SLEDAI score and variables such as microRNA and intracellular protein levels. Spearman's rho for nonlinear distributed variables and Pearson correlation were used for linear distributed variables. The statistical significance threshold was set at *p* < 0.05. All statistical calculations were performed by using the SAS software package, version 9.1 (2002, SAS Statistical Institute, North Carolina).

## 3. Results

### 3.1. Baseline Characteristics of the Study Patients

The baseline characteristics, laboratory data, and microRNA of the SLE patients and healthy controls are listed in Table 1. The age and gender distributions were similar between SLE and normal controls (*p* = 0.07 and 0.37, respectively). The disease activity (SLEDAI-2k) of the 13 lupus patients was 6.08 ± 4.87, with the highest at 17 and lowest at 2. The clinical symptoms of the 13 SLE patients included neurologic involvement in three patients, musculoskeletal involvement in ten patients, hematologic involvement in three patients, renal involvement in one patient, cardiac involvement in one patient, respiratory involvement in two patients, and mucocutaneous involvement in two patients. Six SLE patients had involvement of more than one organ. Overall, these SLE patients were under medication control in a relatively stable disease condition who were regularly followed up with at outpatient clinics. The leukocyte, hemoglobin, c-reactive protein (CRP), liver enzymes, and creatinine levels were similar between the two groups (all *p* > 0.05), which demonstrated the stable and steady state of the SLE patients. The only difference between the two groups was the total cholesterol and the triglyceride levels, which were significantly higher in SLE patients than in normal controls (both *p* < 0.05), but this conferred no clinical significance (comparable statin usage between the two groups, *p* = 0.64) (Table 1).

### 3.2. MicroRNAs Expression in Patients with SLE

The levels of plasma microRNAs were significantly lower in three out of four microRNAs selected in this study (Table 1). Among them, the ΔCT of miR-21-5p, miR-150-5p, and miR221-3p were significantly higher in plasma from SLE patients than in normal controls (all *p* < 0.05, higher ΔCT indicates lower plasma level), except miR-22-3p. The levels of miR-22-3p were similar between SLE patients and normal controls (*p* > 0.05).

### 3.3. Correlations Analysis between MicroRNA and Leukocyte Viral Infection/Activation Markers

**Figure 1:**
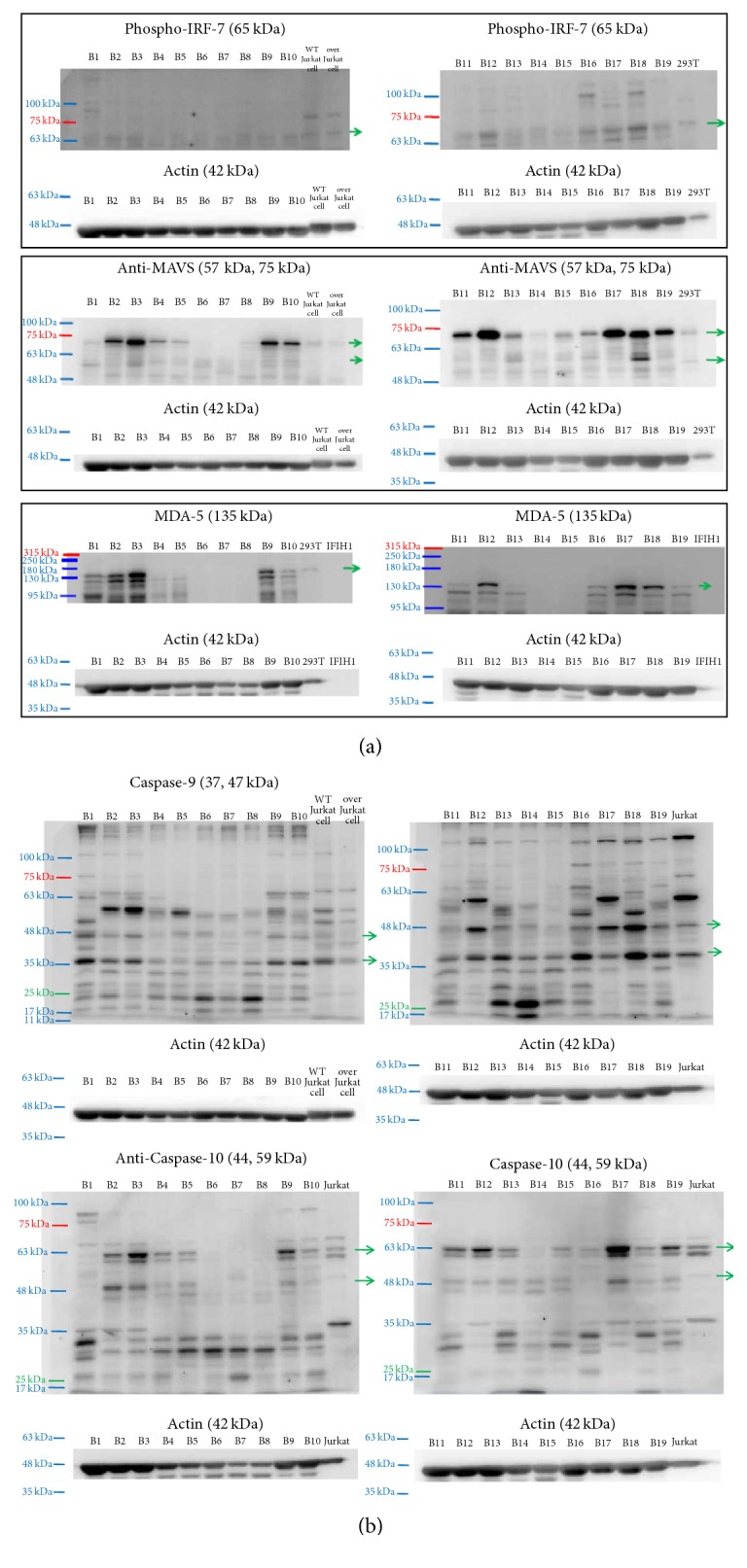
Protein expression in peripheral blood mononuclear cells from systemic lupus erythematosus patients. (a) Intracellular proteins (pIRF7, MAVS, and MDA5) in western blots. (b) Intracellular proteins (caspase-9 and caspase-10) in western blots. pIRF7, phosphorylated interferon regulator factor 7; MAVS, mitochondrial antiviral signaling protein; MDA5, melanoma differentiation-associated protein 5; B1~B19, peripheral mononuclear cell lysate from systemic lupus erythematosus patients.

The association between microRNA and the intracellular protein levels including caspase-9, caspase-10, MDA5, full-length MAVS (70 kD), and mini-MAVS (57 kD), is listed in Table 2, and the western blot data is shown in Figure 1. The ΔCT of miR-150-5p was positively correlated with SLEDAI (*r* = 0.63, *p* = 0.01). The ΔCT of miR-150-5p was also negatively associated with full-length MAVS level (*r* = −0.49, *p* = 0.04, Table 2). Caspase-10 protein levels were negatively associated with plasma miR-22-3p (*r* = −0.47, *p* < 0.05) and miR-21-5p (*r* = − 0.62, *p* = 0.01). Further, the ΔCT of miR-150-5p was positively correlated with CRP (*r* = 0.56, *p* < 0.01).

## 4. Discussion

The present study examined the role of microRNA in mitochondrial apoptotic pathway in SLE, with several major findings. First, the ΔCT of miR-21-5p, miR-150-5p, and miR221-3p were significantly higher in plasma from SLE patients than in normal controls (all *p* < 0.05) (Table 1). Second, the ΔCT of miR-150-5p was positively correlated with both the SLEDAI and CRP. Third, the ΔCT of miR-150-5p is negatively associated with active MAVS and ΔCT of miR-22-3p (*r* = −0.47, *p* < 0.05) and ΔCT of miR-21-5p (*r* = − 0.62, *p* = 0.01) were negatively associated with caspase-10 protein levels.

There is accumulating evidence about the role of microRNA in the pathogenesis of SLE and autoimmune diseases. For example, mir-150 regulates various immune cells, including B cells, T cells, and NK cells [34], and it is a biomarker in lupus nephritis [35], but its expression does not differ in T cells from lupus patients and normal controls [21]. On the other hand, this miRNA is downregulated in skin with psoriasis [20] but increased in plasma during osteogenesis [36] and scleroderma [34] or after a 10-km race [37]. These results suggest that microRNA expression and effects could be tissue-specific and that plasma microRNA reflects a general condition of a patient.

This study linked mir-150-5p with intracellular MAVS protein expression. The MAVS protein levels were significantly higher in patients with lupus than in normal controls and were negatively correlated with lupus activity in our previous study [30]. The dynamic changes of mir-150-5p relating to lupus activity are worthy of further investigation.

**Figure 2:**
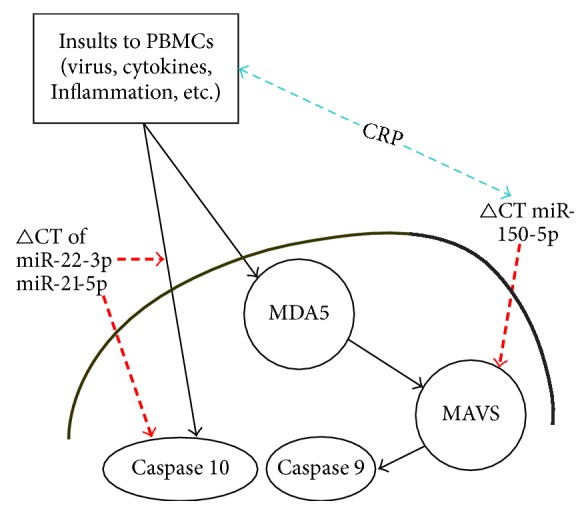
The simplified link between ΔCT of each microRNA and apoptotic molecules in peripheral blood mononuclear cells (PBMCs) in our study. Bidirection (blue) arrow-head thin dash-line: positive correlation between ΔCT microRNA and serum protein; arrow-head (red) bold dash-line: negative correlation between ΔCT microRNA and intracellular protein levels. CRP, C-reactive protein; MAVS, mitochondrial antiviral signaling protein; MDA5, melanoma differentiation-associated protein 5; PBMCs, peripheral blood mononuclear cells.

Cell catabolism is upregulated in SLE, and several recent articles have mentioned apoptosis and microRNAs in SLE [19, 20, 23, 38]. Wang et al. showed that miR221/222 downregulates caspase-10* in vitro* [23]. As seen in Table 2, we noted that caspase-10 is negatively associated with ΔCT of several plasma microRNAs, including miR-22-3p and miR-21-5p. This observation suggests the activation of the extrinsic apoptotic caspase-10-related pathway in SLE [22, 39] and that caspase-10 is influenced by microRNA, which could be reflected by plasma microRNA levels in this study. Furthermore, we previously demonstrated that caspase-10 positively correlates with pIRF7, and caspase-9 and caspase-10 both positively correlate with each other, pIRF7, and MAVS 70 kD [30], indicating that interferons are links to cellular apoptosis and anti-virus immunity in SLE [16, 40–42]. The pathways of MAVS and caspase-10 were examined by detecting plasma microRNA in SLE (Figure 2).

A new direction of SLE studies in microRNA shed light on SLE pathogenesis [43]. Our preliminary study adds several microRNAs that were significantly different from normal controls to the list of previously identified microRNAs. It is worth mentioning that miR-150-5p levels were positively correlated with clinical inflammatory indicator CRP level and the SLEDAI but were negatively associated with MAVS. The idea that interplay between disease activity and infectious disease could be linked by microRNA has been mentioned in other studies [34, 44].

This study had several limitations. First, this was a cross-sectional observational study. More detailed studies are required to determine the real function of plasma microRNA levels in lupus. The concentration of plasma microRNA is low and it needs delicate handling during experiment procedure, and six of our patients' plasma microRNAs were either undetectable or with poor quality which prevent further analysis. A longitudinal study is also required to detect the trend of plasma microRNA in lupus and could reduce variance and improve our ability to predict the prognoses. Second, the case number in this study was small. The difference data number between the plasma microRNA and the intracellular protein levels was due to experiment difficulty of the retrieving plasma microRNA. Large-scale prospective and longitudinal studies are needed to evaluate the prognostic contribution of microRNAs on clinical outcome.

Our study confirmed the hypothesis that these microRNAs were associated with the mitochondrial apoptotic pathway in SLE. MiR-150-5p ΔCT was positively associated with SLE disease activity and was negatively correlated with MAVS 70 kD. The level of microRNA concentration is reversed to the ΔCT, so it is suggesting that this miR-150-5p is positively correlated with MAVS 70 kD and might facilitate anti-viral activity during viral infection and this might be reflected by elevation of CRP levels clinically. The miR-150-5p could be one useful marker demonstrating virus related lupus disease flare-up clinically (Figure 2). On the other hand, miR-22-3p ΔCT and miR-21-5p ΔCT were negatively correlated with caspase-10 levels, where these microRNAs may associate increased extrinsic apoptosis and decreased cell survival, which could reflect monocyte activation-induced cell death.

In conclusion, the plasma microRNA could be a maker demonstrating complex immune milieu in lupus. Some specific microRNA markers could be useful makers for differentiating intracellular immune pathways, such as miR-150-5p in MAVS pathway and miR-22-3p and miR-21-5p in extrinsic apoptosis pathway.

All the underlying research materials related to our article can be accessed on demand by email notification.

## Figures and Tables

**Table 1:** Demographic clinical data of SLE patients and healthy controls.

	Normal controls	SLE	*p* value
	(*n* = 29)	(*n* = 13)	
Age (year)	57.76 ± 5.47	51.31 ± 11.10	0.07
Leukocytes (×1000/ml)	5.53 ± 1.40	6.68 ± 2.24	0.11
Hemoglobin (mg/dL)	13.37 ± 1.66	12.44 ± 1.74	0.12
Hematocrit (%)	40.57 ± 3.75	37.40 ± 4.71	0.05
c-reactive protein (mg/dL)	1.75 ± 2.29	5.98 ± 7.54	0.23
Aspartate aminotransferase (U/dL)	23.88 ± 7.63	40.88 ± 56.02	0.42
Alanine aminotransferase (U/dL)	24.65 ± 17.12	19.13 ± 8.36	0.39
Total cholesterol (mg/dL)	196.9 ± 26.89	221.57 ± 34.07	<0.05^*∗*^
high-density lipoprotein (mg/dL)	59.86 ± 14.85	67.17 ± 19.41	0.31
low-density lipoprotein (mg/dL)	118.62 ± 23.05	117.83 ± 26.23	0.94
Triglyceride (mg/dL)	102.83 ± 52.50	191.71 ± 86.69	0.04^*∗*^
Creatinine (mg/dL)	0.71 ± 0.15	0.86 ± 0.49	0.37
Gender (female: male)	21:08	11:02	0.47
Use of statins (yes: no)	03:26	01:12	0.64
plasma microRNA: miR-541a as control (CT)			
miR-22-3p ΔCT	6.24 ± 1.51	6.36 ± 0.84	0.79
miR-150-5p ΔCT	7.07 ± 1.60	8.71 ± 1.46	<0.01^*∗*^
miR-221-3p ΔCT	5.88 ± 2.19	7.65 ± 1.56	0.01^*∗*^
miR-21-5p ΔCT	2.55 ± 1.43	4.10 ± 0.92	<0.01^*∗*^

SLE, systemic lupus erythematosus; §, data presented with mean ± SD (standard deviation); continuous variables between two groups were compared using Student's *T*-test, between *α*, healthy group, and *β*, SLE; Gender and use of statins were compared using Fisher's exact test; *∗* indicates *p* value < 0.05.

**Table 2:** Correlation analysis between microRNA and leukocyte viral infection/activation markers.

*n* = 13	SLEDAI	casp-9 (37 kD)	casp-10 (59 kD)	MDA5	MAVS (70 kD)	MAVS (57 kD)	CRP
miR-22-3p ΔCT							
*r*	−.12	−.08	−.47^*∗*^	.08	.10	−.38	−.09
*p*	.63	.75	<.05^*∗*^	.77	.69	.13	.65

miR-150-5p ΔCT							
*r*	.70^*∗*^	−.03	−.13	−.36	−.49^*∗*^	−.06	.56^*∗*^
*p*	.00^*∗*^	.91	.60	.15	.04^*∗*^	.81	<.01

miR-221-3p ΔCT							
*r*	−.12	−.12	−.02	.13	.21	−.39	.09
*p*	.65	.64	.93	.61	.40	.11	.65

miR-21-5p ΔCT							
*r*	.02	−.23	−.62^*∗*^	.01	−.07	−.21	.23
*p*	.92	.35	.01^*∗*^	.98	.78	.40	.21

Method: Spearman's rho for nonlinear distributed variables or Pearson correlation for linear distributed variables; *r*, correlation coefficient; *p*, *p* value; *n*, number; casp, caspase; SLEDAI-2K, systemic lupus erythematosus disease activity index 2000; ΔCT, compared with miR-541a as control (CT); pIRF7, phosphorylated interferon regulator factor 7; MAVS, mitochondrial antiviral signaling protein; MDA5, melanoma differentiation-associated protein 5; CRP, c-reactive protein (mg/dL); *∗*, correlation is significant at the 0.05 level.
